# Targeting insulin-like growth factor axis in hepatocellular carcinoma

**DOI:** 10.1186/1756-8722-4-30

**Published:** 2011-07-05

**Authors:** Jennifer Wu, Andrew X Zhu

**Affiliations:** 1 Division of Hematology and Medical Oncology, NYU Cancer Institute, NYU School of Medicine, New York, NY, 10016, USA; 2 Division of Hematology and Medical Oncology, Massachusetts General Hospital Cancer Center, Harvard Medical School, Boston, MA, 02114, USA

## Abstract

The insulin-like growth factor (IGF) axis contains ligands, receptors, substrates, and ligand binding proteins. The essential role of IGF axis in hepatocellular carcinoma (HCC) has been illustrated in HCC cell lines and in animal xenograft models. Preclinical evidence provides ample indication that all four components of IGF axis are crucial in the carcinogenic and metastatic potential of HCC. Several strategies targeting this system including monoclonal antibodies against the IGF 1 receptor (IGF-1R) and small molecule inhibitors of the tyrosine kinase function of IGF-1R are under active investigation. This review describes the most up-to-date understanding of this complex axis in HCC, and provides relevant information on clinical trials targeting the IGF axis in HCC with a focus on anti-IGF-1R approach. IGF axis is increasingly recognized as one of the most relevant pathways in HCC and agents targeting this axis can potentially play an important role in the treatment of HCC.

## Introduction

Hepatocellular carcinoma (HCC) is the 5^th ^most common neoplasm worldwide with more than 600,000 cases per year and the 3^rd ^leading cause of cancer-related death [1,2]. For the past 3 decades, the incidence of HCC in the US has tripled, yet the 1 year survival rate of HCC remains less than 50% [3]. Currently sorafenib is the only medication that shows overall survival advantage compared to placebo in patients with advanced HCC [4,5]. However, the benefits with sorafenib are moderate and its toxicities can be challenging to manage. For patients who fail or cannot tolerate sorafenib, there are currently no standard treatments. Therefore, there is an urgent need to search for novel effective therapies in advanced HCC. Recently, the insulin-like growth factor (IGF) axis has emerged as an important pathway in the development and progression of HCC and as a potential therapeutic target.

Here we review the complexity of IGF axis, the supporting preclinical and clinical data highlighting the significance of this pathway in HCC, and the early clinical trials of targeting this axis in advanced HCC.

## Components of IGF Axis

The insulin-like growth factor (IGF) pathway has highly conserved function in mammals and plays a critical role in energy metabolism and cell renewal in response to nutrients [6-11]. IGF pathway is not only involved in cell growth in tissue culture [12,13], but it also promotes cell proliferation, migration and transformation into malignant clone [12,14]. The IGF-1 pathway revolves around 4 essential components.

### (1) Ligands

The first component contains the IGF ligands, which include both insulin-like growth factor 1 (IGF-1) and IGF-2. Their names are based on the observation that both IGF-1 and IGF-2 are peptides, similar to insulin, and they share 40% homology with proinsulin [15,16]. They are, however, slightly different from insulin structurally by containing an additional domain, which could account for their dramatically different role in neoplasms in comparison with insulin [16].

### (2) Receptors

**Figure 1:**
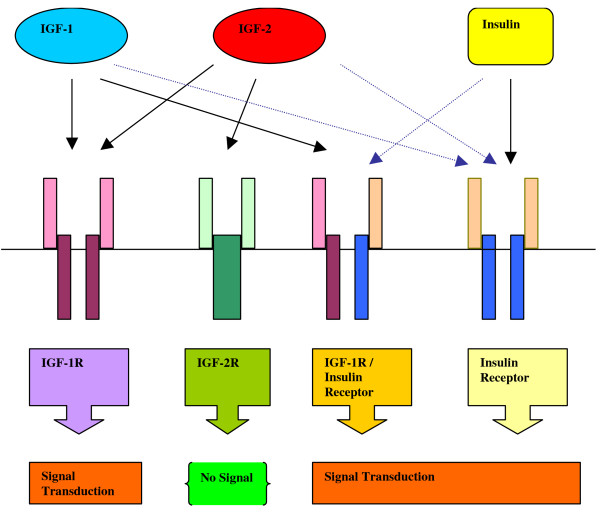
**Binding of insulin and IGF ligands to their receptors**. Insulin receptor and IGF-1 receptor are both tyrosine kinases. IGF-2R functions as a clearance site for IGF-2. Insulin receptor and IGF-1R are homologous and form hemireceptors. IGF-1 binds to IGF-1R and to IGF-1R/Insulin Receptor hemireceptor; it binds to insulin receptor only at very high concentrations. IGF-2 binds to IGF-1R, IGF-2R and binds to insulin receptor only during early fetal development. Insulin binds to insulin receptor, and it binds to IGF-1R/Insulin Receptor hemireceptor at high concentration. Signal transduction is activated after the activation of IGF-1R, IGF-1R/Insulin Receptor hemireceptor and insulin receptor; however, IGF-2R activation results in no signal downstream. Solid lines represent high affinity binding, dotted lines indicate weak binding.

The IGF ligands bind to the second component of the IGF axis, the receptors which include IGF-1 receptor (IGF-1R), IGF-2 receptor (IGF-2R), insulin receptor and hybrid receptors consisting of IGF-1R and insulin receptor hemireceptors (IGF-1R/insulin receptor) (Figure 1). IGF-1 and IGF-2 both bind to IGF-1R with high affinities, and IGF-2 is the only ligand for IGF-2R [6,12,15]. IGF-1 only binds to insulin receptor at extremely high doses, as IGF-1 has 100 fold higher affinity for IGF-1R compared to insulin receptor [16]. IGF-2 usually binds to insulin receptor during fetal development, as later in development when IGF-1R is expressed, IGF-2 binds to IGF-1R more tightly [16,17]. Each IGF-1R/insulin receptor hemireceptor only contains one α and one β subunit; IGF-1 is the preferred ligand for IGF-1R/insulin receptor hybrid receptors compared to insulin, as IGF-1 can tightly bind in the presence of only one α subunit of the hemireceptor, while insulin requires two β subunits of the hemireceptor to provide optimal binding [16].

### (3) Substrates

The third component of the IGF axis refers to the insulin receptor substrate (IRS) and Shc proteins, which are the major signals downstream of IGF-1R activation [16]. There are 4 types of IRS and the important ones include IRS-1 and IRS-2.

### (4) Ligand Binding Proteins

The last key component of the IGF axis consists of IGF binding proteins (IGFBPs). There are 6 members of IGFBPs with high affinities for IGF-1 and IGF-2. For instance, IGFBPs 1-4 bind both IGF-1 and IGF-2 with similar affinities, yet IGFBP-5 and 6 strongly prefer IGF-2 as their ligand.

## Physiologic Functions of IGF Ligands and Receptors

### (1) IGF Ligands

#### (A) IGF-1

The majority of IGF-1 is synthesized in the liver under the influence of growth hormone, which is a major promoter of postnatal growth [18]. However, deletion of liver specific IGF-1 gene in mice showed no difference in growth compared to wild type animals, even though serum IGF-1 level was reduced by 75% [18-20]. Such observations came with no surprise when later on IGF-1 was found to be produced in other organs such as the kidneys, muscle and bone [16]. IGF-1 can act as an autocrine, paracrine or endocrine growth factor, therefore even minimal amount of IGF-1 could still exert its function on postnatal growth [18-20]. Nutrition depletion reduced IGF-1 levels and risk of cancer [12,21], whereas infusion of IGF-1 abolished the protection against carcinogenesis provided by dietary restriction [22]. Epidemiology studies also indicate that IGF-1 is involved in the risk of cancer development. Several studies suggest that height and weight at birth are proportional to the level of IGF-1 in the umbilical cord, and that infants with higher percentile of height and weight at birth tend to develop more common cancers such as breast, prostate and colorectal later in life [22-28].

#### (B) IGF-2

IGF-2 shares 60% homology with IGF-1. Similar to IGF-1, it is also mostly produced in the liver [6,16] and acts in an autocrine, paracrine and endocrine fashion. It is abundant in fetal development, yet its quantity sharply diminishes after birth [16]. IGF-2 knockout mice develop normally except all of them have stunted growth after birth [16], indicating that IGF-2 is critical in growth.

### (2) IGF Receptors

#### (A) IGF-1 Receptor

##### The effects of IGF-1R on apoptosis and cell mobility

Both IGF-1 and IGF-2 bind to IGF-1 receptor 1(IGF-1R), a tyrosine kinase that is structurally similar to insulin receptor (IR) (Figure 1). After IGF ligand binding, the β subunit of IGF-1R undergoes conformational change which causes autophosphorylation of its own tyrosine kinase domain, which leads to the full activation of IGF-1R. IGF-1R induces anti-apoptosis and increases tumor cell mobility. The anti-apoptotic property of IGF-1R was shown in its response to p53, the tumor suppressor gene that promotes apoptosis. Wild type p53 expression inhibited the gene expression of IGF-1R, while mutant p53 increased the gene expression of IGF-1R [16,29]. Oncogenes such as Src kinase and Akt kinase both stimulated the gene expression of IGF-1R, providing more evidence that IGF-1R is vital in carcinogenesis [16,29-31]. In addition, IGF-1R also stimulates cell mobility, as demonstrated by its activity in melanoma cell lines [32].

##### IGF-1R and malignant transformation

Another important role of IGF-1R in carcinogenesis is its ability to transform and maintain the transformed phenotype [33]. Mouse embryo fibroblasts possess an extremely strong tendency to spontaneously transform in culture without any additional factors [33], which was no surprise given IGF-1R overexpressed in mouse embryo fibroblasts led to transformation [33-36]. However, when IGF-1R gene in mouse embryo fibroblasts was disrupted, these fibroblasts failed to transform, even in the presence of the most potent oncogenes such as SV40 T antigen, Ha-ras oncogene and activated c-Src [33,37-41]. An even more noteworthy observation was that when IGF-1R was reintroduced, these mouse embryo fibroblasts again restored their ability to rapidly transform.

##### Toxicities of IGF-1R inhibition

IGF-1R is required for anchorage independent growth, and inhibition of IGF-1R causes apoptosis without toxicities in vivo. Human prostate cancer cells usually form anchorage independent growth, however; when IGF-1R was abolished, these cells failed to grow in culture, and the same model showed no tumor formation in mice [33,42-45]. These observations indicate that IGF-1R is an essential requirement for anchorage independent growth, a pattern common in cancer cell proliferation. In animal models with transformed tumors where IGF-1R was overexpressed, strategies that caused IGF-1R downregulation such as antisense against IGF-1R produced profound tumor apoptosis and massive reductions of metastases [46,47]. Interestingly, IGF-1R is not required for normal cell growth, as its absence provided no growth inhibition on monolayer cell culture [45], eluting to the possibility that anti-IGF-1R strategies could produce minimal side effects on normal tissues.

#### (B) IGF-2 Receptor

There are no known downstream signals related to IGF-2R activation and it appears that IGF-2R mainly serves as a clearance site for its only ligand, IGF-2 [6]. Most of the effects of IGF ligands are mediated through IGF-1R, a transmembrane tyrosine kinase [6,12,16].

#### (3) IGF-1R Substrates

**Figure 2:**
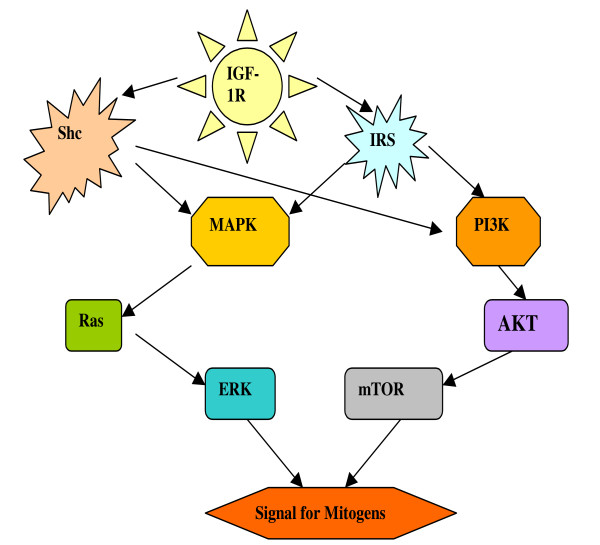
**IGF-IR downstream signal transduction**. The activated IGF-IR initiates signalling through two separate connections, the insulin receptor substrate (IRS) and the Shc proteins. Both IRS and Shc proteins can in turn activate both MAP Kinase (MAPK) and PI3 kinase (PI3K) pathways. MAPK pathway leads to activation of Ras and then ERK, and PI3K pathway activates AKT/mTOR, both then stimulate signals for mitogens.

Among the substrates of IGF-1R, IRS plays a prominent role in exerting the activity of IGF-1R by activating downstream signals [16]. After IGF-1R activation, additional tyrosine residues are then phosphorylated, which act as docking stations for substrates such as the insulin receptor substrate (IRS) and Shc adaptor proteins (Figure 2). IRS and Shc adaptor proteins then recruit additional factors to yield activations of two major cascades, the phosphatidyl inositol 3-kinase (PI3K) and the mitogen-activated protein kinase (MAPK), both result in cell differentiation, proliferation and anti-apoptosis [16,22]. There are currently 4 types of IRS proteins [48], the effects of IRS-1 and 2 are opposite to that of IRS-3 and 4 [16]. IRS-1 is the most well understood IRS, and it is essential to the activation of IGF-1R. When IRS-1 was abundant, it promoted cell size growth, activated p70 ^S6K^, a kinase that promotes cell proliferation and leads to transformation [49]. Meanwhile, IRS-1 turned off IGF-1R's stimulation for differentiation through its phosphotyrosine binding (PTB) domain, therefore inhibited differentiation and stimulated transformation [50]. When IRS-1 was inhibited or malfunctions, such as the case when there was a mutation of its PTB domain, transformation no longer continued and these cells tend to undergo differentiation. The inhibitor of p70^S6K ^such as rapamycin, which is an inhibitor in the mammalian target of rapamycin (mTOR) pathway, also produced similar effects as the mutated PTB domain, thus cells exposed to rapamycin tend to grow slowly with good differentiation [51].

#### (4) IGF Binding Proteins

##### (A) IGFBP-3

One of the key regulators of IGF expression is the family of IGF Binding Proteins (IGFBPs) [6]. The predominant form of IGFBPs is IGFBP-3, which comprises of 90% of all IGFBPs in serum [15,16], and it binds to the majority of circulating IGF-1 and IGF-2. IGFBPs that include IGFBPs 1, 3, 4 and 6 usually limit IGF access to IGF-1 receptor, therefore decrease the availability of IGFs and diminish their effects on cancer progression [6].

##### (B) Other IGFBPs

Other IGFBPs such as IGFBP-2 and 5 seem to increase the bioavailability of IGF ligands, therefore play an opposite role of IGFBP-3 [6]. Both in vitro and in vivo evidence support the observation that antisense strategy targeting IGFBP-2 or 5 decreases neoplastic growth [6,52].

### Evidence of IGF Axis Involvement in Hepatocarcinogenesis

#### (1) Role of IGF Ligands

##### (A) IGF-1

In human HCC tissues, IGF-1 mRNAs were expressed at lower levels than the surrounding normal liver tissues [18,53]. This could be related to the observation that growth hormone receptor level was low in HCC tissues [18,53], and growth hormone stimulation thus was low, and the downstream signals such as IGF-1 level would be accordingly low.

##### (B) IGF-2

##### IGF-2 overexpression and its effects on apoptosis and angiogenesis in HCC

IGF-2 has been reported to be overexpressed in animal models of hepatocarcinogenesis and in human HCC [50,54-60]. IGF-2 has been linked to carcinogenesis by providing a stimulatory effect on cell proliferation and angiogenesis, both critical in HCC development. In a study using 2 human HCC cell lines, high levels of IGF-2 were demonstrated, and anti-sense oligonucleotides used to target IGF-2 mRNA showed reduction of IGF-2 mRNA and protein levels, which corresponded to a remarkable decrease in cell proliferation [18,61]. In a study of molecular profiling of human HCC samples, overexpression of IGF-2 was related to a cluster of gene signature that downregulates apoptosis [62], indicating a potent anti-apoptotic effect of IGF-2. The relationship between IGF-2 and angiogenesis was demonstrated in human HCC cell cultures. Under hypoxia environment, IGF-2 mRNA levels in human HCC tissue increased, and IGF-2 overexpression directly increased vascular endothelial growth factor (VEGF) mRNA and protein levels [63]. It suggested a pro-angiogenic effect of IGF-2, an important pathway in HCC development and metastasis.

##### Animal models of IGF-2 and preneoplastic lesions for HCC

In rodents, diethylnitrosamine (DEN) induced 100% development of glycogen rich hepatic lesions, which are precursors to HCC, and up to 98% of such lesions expressed IGF-2 mRNA [54,64]. These results highlight a vital role of IGF-2 early in hepatocarcinogenesis. The expression of IGF-2 has been shown to be a common pathway leading to hepatocarcinogenesis regardless of the species or the process of HCC development [65]. In transgenic mice where IGF-2 levels were persistently 20 times higher than normal control mice, a diverse spectrum of tumors were seen at a much higher frequency than the controls, and HCC was the most common malignancy by 18 months of age [66].

##### Re-emergence of fetal IGF-2 expression in human HCC

The expression of IGF-2 is very unique in fetal development, as it is maternally imprinted; therefore it is monoallelic [6,18]. In adults, IGF-2 becomes biallelic [18]. In fact, IGF-2 overexpression in HCC showed re-emergence of fetal IGF-2 by the identification of fetal promoter activation [67]. In all 15 samples of human HCC tested in a study, the overexpression of maternally imprinted fetal IGF-2 was demonstrated [51]. In a study from Hong Kong, 30 HCC samples from patients examined using northern blot analysis showed more than 93% of the adult promoter IGF-2 transcripts were repressed, while 93% of the adult type IGF-2 transcripts were detected in nontumourous tissues [68,69].

##### The Interaction of IGF-2 with HCC risk factors

The importance of IGF-2 in HCC development is further demonstrated in its relationship with risk factors of HCC such as hepatitis B and C [70]. In patients with chronic hepatitis C and cirrhosis, the overexpression of IGF-2 was clearly related to hepatitis C viral replication [71]. In patients with chronic hepatitis B, HBV X protein stimulated IGF-2 expression by binding to the fetal promoter of IGF-2, therefore directly stimulating fetal transcript expression of IGF-2 in HCC [72]. Furthermore, aflatoxin has been shown to be synergistic with hepatitis B in the carcinogenesis of HCC, and p53 gene mutation induced by aflatoxin increased the expression of IGF-2 in HCC patients with hepatitis B infection [73].

#### (2) Role of IGF Receptors

##### IGF-1R overexpression in vitro in HCC

In a study where 10 HCC cell lines (including PLC HCC cell line) were tested, all of them showed elevated IGF-1R mRNA [50]. Furthermore, the addition of both IGF-1 and IGF-2 to the PLC HCC cell line induced increased cell proliferation in a dose dependent manner, showing that the major tumor promoting effects of IGF ligands on HCC are exerted through IGF-1R [46].

##### IGF-1R overexpression in animal models of hepatocarcinogenesis

In a model utilizing pancreatic islet transplantation into the livers of diabetic rats, a well established series of events led to development of HCC from preneoplastic foci [74]. When HCC developed from preneoplastic foci in this animal model, the expression of IGF-1R significantly increased, which could explain the phenomenon that the increase in mitotic activity was more than the increase in the rate of apoptosis [18,75]. IGF-1R is therefore crucial in both the development of and the growth of HCC, making IGF-1R an ideal target in the treatment of HCC.

##### The Inhibitory effects of IGF-2R on IGF-1R

IGF-2R is closely associated with transforming growth factor β (TGF-β), a very potent growth inhibitor [76]. For instance, in human HCC tissues, the levels of both TGF-β and IGF-2R protein were reduced compared to those in adjacent normal liver tissues [66]. The expression of IGF-2R was significantly lower in several HCC cell lines in vitro, in HCC animal models and in human HCC tissues [77,78]. The role of IGF-2R in IGF axis appears to serve as a site for IGF-2 clearance, therefore reduces the availability of a potent ligand for IGF-1R, the major gateway for carcinogenesis, tumor growth and proliferation. IGF-2R therefore provides an indirect inhibitory effect on IGF-1R.

#### (3) Role of IGF Substrates

##### (A) IRS-1

The overexpression of IRS-1 has been described in human HCC cell lines and tissues [79]. IRS-1 leads to activation of downstream mitogens such as PI3K and MAPK. In human HCC cell lines, IRS-1 developed acquired resistance to apoptosis, indicating a potent role of IRS-1 in the promotion of continued cell growth in HCC [79].

##### (B) IRS-2

IRS-2 is a major downstream signal of insulin pathway in the liver, and its function in hepatocarcinogenesis is demonstrated in animal models. When SV40 large T antigen or DEN was applied in murine models, IRS-2 overexpression was detected in both preneoplastic foci and HCC lesions, with higher levels in HCC nodules [47]. A similar observation was reproduced in human HCC cell lines and tissue specimens, suppression of IRS-2 levels led to increased apoptosis. Together with IRS-1, IRS-2 also contributes to hepatocarcinogenesis, as demonstrated by its early emergence in preneoplastic lesions, and its anti-apoptotic property. IRS-1 and 2 therefore create an optimal environment for HCC growth.

#### (4) Roles of IGFBPs

##### (A) IGFBP-3

In a study comparing IGFBP-3 levels in human normal liver, cirrhotic liver and HCC, the expression of IGFBP-3 mRNA levels was significantly reduced in HCC [80]. In a human HCC cell line, addition of exogenous IGFs stimulated mitosis, but this mitogenic effect was greatly reduced by IGFBP-3 [46]. Furthermore, addition of recombinant human IGFBP-3 induced growth inhibition of the human HCC cell lines HepG2 and PLC [81]. The role of IGFBP-3 on tumor growth inhibition can be further explained by IGFBP-3's induction by p53, a tumor suppressor gene essential in apoptosis and cell cycle arrest [15].

##### (B) IGFBP-7

In a study examining radiation induced HCC mouse model, northern analysis showed decreased expressions of IGFBP-7 (a low affinity IGFBP) in HCC compared to normal liver tissues, which was inversely related to anchorage-independent growth in HCC cell lines [82]. A similar trend of reduced IGFBP-7 level was seen in human HCC tissues. When IGFBP-7 cDNA was injected to radiation induced HCC mouse model, the volume of HCC was greatly reduced. IGFBP-7, although has relatively low affinity toward IGF-1 and IGF-2, exerts a similar anti-tumor effect as its high affinity IGFBP counterpart IGFBP-3.

##### (C) IGFBP protease inhibitors

Metalloproteinase belongs to IGFBP proteases that degrade IGFBP-3. In a transgenic murine HCC model overexpressing the inhibitor of metalloproteinase (TIMP1), IGFBP-3 degradation was reduced, and serum level of IGFBP-3 was subsequently increased, which decreased the bioavailable IGF-2 ligand and its downstream signalling. This resulted in reduced liver hyperplasia, despite the activation of IGF-2 by a strong oncogene such as SV40 T antigen [83]. It provided evidence that IGFBP proteases and IGFBPs are equally important in the regulation of IGF ligand bioavailability and their downstream effects on IGF axis activation.

### Targeting IGF System and Early Clinical Trials

There are several strategies in the therapeutic considerations involving IGF axis in the treatment of HCC and other tumors. The first method targets the ligand to reduce its activity, the second inhibits the function of the receptor, and the third modulates the downstream signals of IGF-1R pathways (Table 1).

**Table 1:** Agents in clinical development that target the insulin-like growth factor pathway

Company	Compound	Mechanism of Action	Phase of Clinical Development	Dosing	Types of Cancers Tested
MedImmune	MDI-573 [89]	Fully human monoclonal antibody of IGF-1 and IGF-2	I	IV every 3 weeks	Solid tumors

Merck	MK-0646 [90]	Monoclonal antibody of IGF-1R	II	IV weekly, 3 weeks on, 1 week off	Non-small cell lung cancer, small cell lung cancer, prostate, breast, pancreas

Imclone	IMC-A12 [91,99]	Fully human Monoclonal antibody of IGF-1R	II	IV every 1 or 2 weeks	HCC, Colorectal, pancreas, mesothelioma, thymoma, prostate, head and neck

Biogen-Idec	BIIB 022 [92]	Monoclonal antibody of IGF-1R	I/II	IV every 2 weeks	HCC, non-small cell lung cancer

Sanofi-Aventis	AVE 1642 [98]	Humanized antibody of IGF-1R	I	IV every 3 weeks	HCC, multiple myeloma

Roche	R1507 [118]	Fully human IgG1 recombinant antibody of IGF-1R	I	Every 1 or 3 weeks	Solid tumors and Lymphoma

Amgen	AMG 479 [119]	Fully human Monoclonal antibody of IGF-1R	II/III	IV every 2 weeks	Pancreas, colorectal, Ewing's sarcoma, ovarian

Pfizer	CP-751871 [93]	Fully human Monoclonal antibody of IGF-1R	II/III	IV every 3 weeks	Non-small cell lung cancer

OSI	OSI-906 [100]	Small molecule inhibitor of IGF-1R	II/III	Oral twice a day	Adenocortical carcinoma, Ovarian

Novartis	AEW54, ADW742 [120,121]	Small molecule inhibitor of IGF-1R	preclinical	NA	NA

BMS	BMS-554417 [102]	Small molecule inhibitor of IGF-1R	preclinical	NA	NA

### (1) Anti-Ligand Approach

One of the first drugs to be tested was somatostatin. However, as it only lowered serum IGF-1 level to a modest degree without achieving desired reduction, it showed no anti-neoplastic activity [84]. Metformin lowered insulin levels in patients with hyperinsulinemic states such as in obesity, a major risk factor for HCC. The reduction of insulin was significant, yet its effect on IGF-1 and IGF-2 was minimal, making metformin a weak candidate in the treatment of HCC [85]. Ongoing studies utilizing growth hormone antagonists or IGF ligand specific antibodies have demonstrated some activity in prostate and breast cancer cell lines, suggesting their potential in the treatment of HCC [86,87]. MEDI-573 is a first in human neutralizing antibody against both IGF-1 and IGF-2, has shown promising activity in vivo based on its inhibition of downstream IGF signalling [88], and is now being tested in phase I solid tumors.

### (2) Anti-Receptor Approach

#### (A) Monoclonal antibodies of IGF-1R

##### Single agent activity of monoclonal antibodies of IGF-1R in vitro, in vivo and in phase I solid tumors

The majority of anti-IGF strategies focused on IGF-1R, the key component of IGF axis that provides mitogenic signal for tumor growth. The most common strategy utilized is the receptor-specific antibodies. For instance, pharmacodynamic studies of MK-0646 (Merck) on neoplastic tissues demonstrated reduction of phosphorylated AKT and phosphorylated S6 kinase, two downstream targets of IGF-1R. MK-0646 also decreased tumor proliferation as shown by reduction in the proliferation marker Ki67 [89,90]. This observation provided a rationale to use this class of antibodies in the treatment of HCC, and it was supported by additional data generated using IMC-A12 (Imclone), a human monoclonal antibody that blocks IGF-1R, both in vitro and in vivo [91]. In hepatoma cell lines, 2 hour incubation with IMC-A12 completely blocked downstream signalling of IGF-1R as shown by the suppression of phosphorylated AKT and phosphorylated S6 kinase [91]. In addition, 10 day treatment with IMC-A12 in HCC xenografts led to 40% reduction of tumor volume and 40% prolongation of overall survival without additional toxicity compared to control animals [91]. In a phase I study of refractory solid tumors using IMC-A12 as a single agent, a patient with HCC had stable disease for up to 9 months [92].

##### Monoclonal antibodies of IGF-1R in combination with chemotherapy in Phase II and III studies

One of the most studied IGF-1R antibodies is CP-751871 (Pfizer) and it showed rather promising activity in a phase II study in patients with advanced non-small cell lung cancer. When it was added to carboplatin and paclitaxel as a first line regimen, the response rate increased from 32% to 46%. What was even more impressive was in patients with squamous histology, the response rate was as high as 71% [93]. The most common side effect in this phase II study was hyperglycemia. The subsequent ambitious Phase III study looked at patients with stage IIIB or IV non-small cell lung cancer, and randomized them to receive carboplatin and paclitaxel either with or without CP-751871. This study was halted in late 2009 due to unexpected increase in fatal events in the experimental arm [93], and it could be partially explained by the most common side effect of hyperglycemia. The consequence of IGF-1R inhibition leads to compensatory increase of growth hormone stimulation that promotes liver gluconeogenesis, resulting in hyperglycemia [94]. What we could learn from this surprising result is that there are subsets of patients who could potentially benefit from IGF-1R inhibitors such as CP-751871 [95]. For instance, in the experimental arm, patients with low IGF-1 levels (< 5 pg/ml) before treatment with CP-75871 were more likely to suffer fatal events within 60 days of treatment. The same group of patients also had much shorter median overall survival compared to the ones with higher pretreatment IGF-1 levels (7 months vs. 10.4 months). Conversely, for patients with higher pretreatment IGF-1 levels, those who received the experimental treatment that included CP-751871 had a trend toward higher median overall survival compared to those who received the standard chemotherapy (10.2 months vs. 7 months). Further analysis from the phase II study also showed that IGF-1R was present in the highest level in patients with squamous histology, which could explain the observed high response rate in squamous cell patients who received CP-75871 [96]. Such observation was consistent with a presentation at ASCO GI in 2011, in which data of 288 patients with HCC were analyzed. In this study, pretreatment lower plasma IGF-1 and higher plasma VEGF levels significantly correlated with advanced clinicopathologic parameters and poor overall survival, with an optimal cut off point of 26 pg/mL and 450 pg/mL, respectively. The combination of low IGF-1 and high VEGF predicted median overall survival of 2.7 months compared with 19 months for patients with high IGF-1 and low VEGF (p < 0.0001) [97]. Such information provided insights into the specific patient subsets in HCC where IGF-1 levels would offer additional prognostic significance. Whether baseline plasma IGF-1 levels could be used to predict response to IGF axis inhibition in HCC remains to be explored.

##### IGF-1R monoclonal antibodies in HCC

IMC-A12 was studied as a single agent in patients with advanced HCC as a front line systemic therapy. This study unfortunately was terminated due to futility. The pre-planned primary endpoint of progression free survival rate at 4 months was only 30% and median overall survival of 8 months [98]. Up to 46% of patients developed grade 3-4 hyperglycemia, similar to what was seen in the phase II NSCLC study of CP-751871 [93], thus raising the possibility that hyperglycemia could be the dose limiting toxicity of IGF-1R monoclonal antibodies. Hyperglycemia and its subsequent increase of growth hormone could also contribute to the disappointing activity of this class of drugs. BIIB022 (Biogen-Idec) is an anti-IGF-1R monoclonal antibody that blocks binding of both IGF-1 and IGF-2 to IGF-1R [92]. It does not contain Fc effector function, therefore can potentially minimize toxicities in healthy tissues expressing IGF-1R [92]. This agent does not appear to cause hyperglycemia, a common side effect of receptor specific antibodies [92]. Hyperglycemia has been attributed to insulin resistance secondary to high levels of growth hormone, a compensatory reaction to IGF-1R antibodies [94,95]. The class of IGF-1R monoclonal antibodies share similar side effect profiles, including minimal dose limiting toxicities. These favorable safety profiles make them ideal candidates in the combination therapy with current available chemotherapy or biologic therapy [6]. BIIB022 showed inhibition of tumor growth in HCC cell line HepG2, and this inhibitory effect was enhanced by addition of sorafenib [92], the only FDA approved medication for patients with advanced HCC. A planned phase I/II study comparing sorafenib with or without BIIB022 in patients with advanced HCC was terminated due to a business decision of Biogen-Idec. AVE-1642 (Sanofi-Aventis) is another IGF-1R antibody that was initially studied in advanced HCC patients in a phase I study in combination with sorafenib [99], the study was terminated not related to either efficacy or toxicity concerns. Although IMC-A12 lacks single agent activity in HCC, its combination with sorafenib could potentially yield synergy. It is currently undergoing phase I study in combination with sorafenib in patients with HCC, the result of this clinical trial may help understand the clinical benefits of combining IGFR-1R monoclonal antibodies and sorafenib in HCC.

#### (B) Small molecule inhibitors of IGF-1R

A major advantage of small molecule inhibitor is its ability to inhibit both IGF-1R and insulin receptor. Such ability was demonstrated in several human tumor cell lines, where phosphorylated IGF-1R and its downstream proteins, including ERK and p70^s6k ^were all effectively inhibited by OSI-906 (OSI) [100]. In addition, it inhibited phosphorylated insulin receptor in both primary human hepatocytes and HCC cell line HepG2. IGF-1R and insulin receptor interaction has been seen in many human tumor cell lines after the appearance of IGF-1R monoclonal antibodies. For instance, when IGF-1R phosphorylation was reduced with the treatment of IGF-1R monoclonal antibody, phosphorylated insulin receptor also increased [100].

Even though IGF-1R plays a dominant role in the activation of IGF axis, insulin receptor becomes very important when IGF-1R is blocked, such as the case with IGF-1R monoclonal antibodies. When IGF-1R is blocked, all the IGF-1 and IGF-2 (ligands for IGF-1R) are available to bind insulin receptor. There are 3 ways how insulin receptor activates the IGF axis. First, when IGF-1 levels increase with IGF-1R inhibition, its binding to insulin receptor also increases, which leads to more insulin receptor activation. Second, IGF-2 usually binds to insulin receptor with very low affinity, however; when IGF-2 fetal transcripts are reactivated, such as in HCC, the affinity of IGF-2 for insulin receptor increases dramatically. Additional insulin receptor is therefore turned on through IGF-2. Third, the overexpression of insulin receptor was demonstrated in numerous human cancers including HCC, and its overexpression was linked to tumor growth and cell survival [101].

*BMS-554417*

Several small molecule tyrosine kinase inhibitors of IGF-1R such as BMS-554417 (Bristol-Myers-Squibb) are under development [102-107]. There have been encouraging in vitro and in vivo data in broad range of cancers with activated IGF axis. Current phase I data on drug tolerability will provide more information regarding the feasibility of such medications in the potential treatment for advanced HCC.

*OSI-906*

OSI-906 (OSI) is a potent tyrosine kinase inhibitor of both IGF-1R and insulin receptor. The unique advantage of OSI-906 over previous class of anti-IGF drugs is its ability to minimize the activity of IGF-2 where IGF-1R inhibition alone will not be sufficient. In cancers such as adenocortical carcinoma and HCC, where insulin receptor binds to IGF ligands with higher affinity, OSI-996 is able to inhibit both insulin receptor and IGF-1R to achieve maximum inhibition of the IGF axis [108-110]. A phase III study using OSI-906 in patients with adenocortical carcinoma is ongoing. OSI-906 is therefore considered one of the desirable drugs to be tested in patients with HCC.

### (3) Approach that targets other pathways

AMP-activated protein kinase (AMPK) pathway is one of the upstream signalling pathways above mTOR [6]. The AMPK activation effects are quite complex, and although experimental models of AMPK activators demonstrate their anti-proliferation effects, they could also potentiate cell survival after exposure to stress [110-113]. Additional studies on activators of AMPK are required to understand the role of such class of medications prior to its use as anti-neoplastic agents. Another active downstream signal of IGF axis is the mTOR pathway, which is downstream of PI3K/AKT signal. Everolimus (Novartis) is being studied in patients with sorafenib refractory HCC in a phase III trial. A third active pathway involves MAPK, and inhibitors of this pathway are currently in very early phase of investigation [114].

### (4) Combination Therapies

As most of IGF-1R inhibitor molecules have minimal dose limiting toxicities in phase I studies, and IGF-1R activation reduces responsiveness of antineopalstic therapies [6], it is possible to combine IGF-1R inhibitors with certain chemotherapies. For instance, IGF-1R overexpression has been associated with resistance to epidermal growth factor receptor (EGFR) inhibitors and mTOR inhibitors [115,116]. The idea of combining IGF-1R inhibitors and agents such as erlotinib or everolimus could be a promising strategy in the management of advanced HCC.

## Conclusions

In recent years, the understanding of IGF pathway in cancer has led to development of IGF inhibitors which show promising anti-cancer signals in early phase I studies. According to the World Health Organization, more than 50% of cancers come from countries where obesity is a prominent risk factor, and that cancer mortality now is more than that of tuberculosis, malaria and AIDS combined [117]. In the next 3 decades, the incidence of HCC in the US is expected to be among the fastest growing cancers partly due to the increasing incidence of obesity [6,12]. IGF axis is an essential pathway in the development of hyperinsulinemia, a condition closely related to obesity, which in turn increases the risk for HCC [6]. Agents that target the IGF axis, an active pathway in carcinogenesis and progression of HCC, provide an alternative strategy in the management of HCC. We are only in the beginning era of realizing the complexities of IGF pathway, additional research in the understanding of both basic science and clinical applications of anti-IGF agents will provide insights into the value of IGF inhibition in the treatment of HCC.

## Abbreviations

HCC: hepatocellular carcinoma; IGF: insulin-like growth factor; IGF-1: insulin-like growth factor 1; IGF-2: insulin-like growth factor 2; IGF-1R: insulin-like growth factor 1 receptor; IGF-2R: insulin-like growth factor 2 receptor; IRS: insulin receptor substrate; IGFBPs: insulin like growth factor binding proteins; PI3K: phosphatidyl inositol 3-kinase; MAPK: mitogen-activated protein kinase; PTB: phosphotyrosine binding; mTOR: mammalian target of rapamycin; VEGF: vascular endothelial growth factor; DEN: diethylnitrosamine; TGF-β: tumor growth factor beta; AMPK: AMP-activated protein kinase; EGFR: epidermal growth factor receptor.

## Competing interests

The authors declare that they have no competing interests.

## Authors' contributions

JW contributed to the collection and interpretation of data, helped drafting the manuscript. AXZ conceived and designed the study, provided critical revisions of its intellectual content, and gave final approval of the version to be published. All authors read and approved the final manuscript
